# Hexaaqua­cobalt(II) 4,4′-(1,2-dihy­droxy­ethane-1,2-di­yl)dibenzoate monohydrate

**DOI:** 10.1107/S1600536810037451

**Published:** 2010-09-30

**Authors:** Ping Tang, Dan Ma, Zhan-qing Chen

**Affiliations:** a Energy Engineering College, Xiangtan University, 411100 Xiangtan, People’s Republic of China; b State Key Laboratory for Geomechanics and Deep Underground Engineering, Xuzhou 221008, People’s Republic of China

## Abstract

The title compound, [Co(H_2_O)_6_](C_16_H_12_O_6_)·H_2_O, is composed of one 4,4′-(1,2-dihy­droxy­ethane-1,2-di­yl)dibenzoate anion lying on an inversion center, one [Co(H_2_O)_6_]^2+^ dicationic complex and a solvent water mol­ecule located on mirror planes. In the crystal, a chain is constructed *via* O—H⋯O hydrogen bonds involving the carboxyl­ate and hydroxyl groups of the organic anion; the chains are further connected into a three-dimensional framework by additional O—H⋯O hydrogen bonds between the [Co(H_2_O)_6_]^2+^ cations, solvent water mol­ecules and the anions.

## Related literature

For background to metal-organic structures and their potential applications as functional materials, see: Liang *et al.* (2000 ▶); Kondo *et al.* (2004 ▶); Lin *et al.* (2004 ▶); Fan & Hanson (2005 ▶); Laborda *et al.* (2004 ▶); Fei *et al.* (2005 ▶); Zhang *et al.* (2006 ▶).
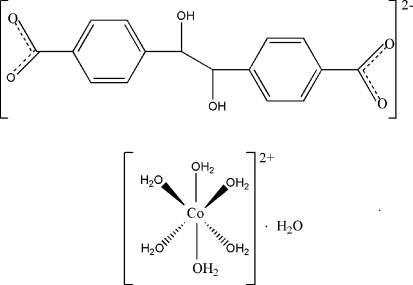


## Experimental

### 

#### Crystal data

[Co(H_2_O)_6_](C_16_H_12_O_6_)·H_2_O
*M* *_r_* = 485.30
Monoclinic, 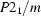

*a* = 6.0430 (6) Å
*b* = 20.487 (2) Å
*c* = 8.6341 (9) Å
β = 104.115 (1)°
*V* = 1036.66 (18) Å^3^
*Z* = 2
Mo *K*α radiation
μ = 0.89 mm^−1^
*T* = 298 K
0.38 × 0.20 × 0.18 mm

#### Data collection

Bruker SMART 1000 CCD area-detector diffractometer
Absorption correction: multi-scan (*SADABS*; Bruker, 2007 ▶) *T*_min_ = 0.728, *T*_max_ = 0.856
5184 measured reflections
1867 independent reflections
1675 reflections with *I* > 2σ(*I*)
*R*_int_ = 0.039

#### Refinement

*R*[*F* ^2^ > 2σ(*F* ^2^)] = 0.075
*wR*(*F* ^2^) = 0.177
*S* = 1.25
1867 reflections
144 parameters
11 restraints
H-atom parameters constrained
Δρ_max_ = 0.58 e Å^−3^
Δρ_min_ = −0.46 e Å^−3^

### 

Data collection: *SMART* (Bruker, 2007 ▶); cell refinement: *SAINT* (Bruker, 2007 ▶); data reduction: *SAINT*; program(s) used to solve structure: *SHELXS97* (Sheldrick, 2008 ▶); program(s) used to refine structure: *SHELXL97* (Sheldrick, 2008 ▶); molecular graphics: *SHELXTL* (Sheldrick, 2008 ▶); software used to prepare material for publication: *SHELXTL*.

## Supplementary Material

Crystal structure: contains datablocks I, global. DOI: 10.1107/S1600536810037451/nk2059sup1.cif

Structure factors: contains datablocks I. DOI: 10.1107/S1600536810037451/nk2059Isup2.hkl

Additional supplementary materials:  crystallographic information; 3D view; checkCIF report

## Figures and Tables

**Table 1:** Hydrogen-bond geometry (Å, °)

*D*—H⋯*A*	*D*—H	H⋯*A*	*D*⋯*A*	*D*—H⋯*A*
O3—H3⋯O1^i^	0.82	2.00	2.811 (8)	168
O1*W*—H1*W*⋯O1^ii^	0.85	1.96	2.814 (9)	180
O1*W*—H2*W*⋯O2^iii^	0.85	1.81	2.665 (8)	179
O2*W*—H3*W*⋯O3^iv^	0.85	2.00	2.847 (8)	180
O2*W*—H4*W*⋯O5*W*^v^	0.85	2.19	3.035 (11)	179
O3*W*—H5*W*⋯O4*W*^vi^	0.85	1.93	2.778 (11)	172
O3*W*—H6*W*⋯O5*W*	0.85	1.91	2.756 (13)	171
O5*W*—H9*W*⋯O2	0.85	1.93	2.767 (10)	169
O4*W*—H7*W*⋯O1^vii^	0.84	1.88	2.695 (7)	163
O4*W*—H7*W*⋯O2^vii^	0.84	2.70	3.296 (9)	130

Symmetry codes: (i) 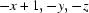; (ii) 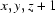; (iii) 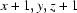; (iv) 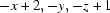; (v) 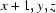; (vi) 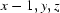; (vii) 
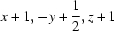
.

**Fig. 1.**
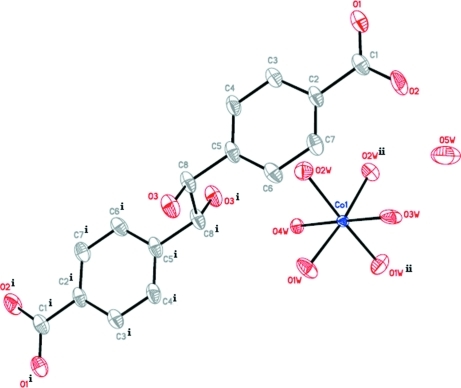
The title compound, with the atom-numbering scheme. Non-H atoms are shown with 30% probability displacement ellipsoids (H atoms are omitted for clarity). [Symmetry codes: (i) 2-x, -y, 1-z; (ii) x, 0.5-y, z.]

**Fig. 2.**
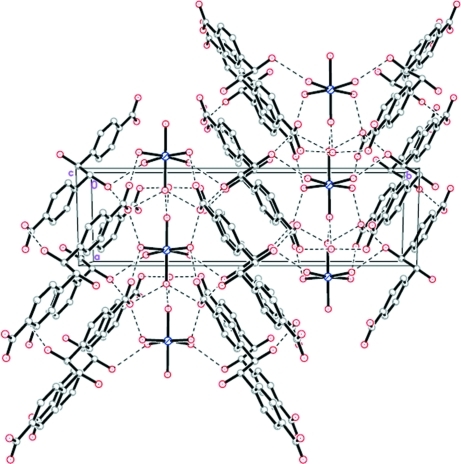
The packing and hydrogen bonding of the title compound down the a axis (H atoms is not shown in the picture for clarity).

## supplementary crystallographic information

### Comment

Metal-organic coordination polymers have been greatly developed in recent years due to their captivating structure (Kondo *et al.*, 2004; Fan *et al.*, 2005) and potential applications as functional materials in electronic (Lin *et al.*, 2004), magnetic (Laborda *et al.*, 2004; Liang *et al.*, 2000; Fei *et al.*, 2005) and optical (Zhang *et al.*, 2006) fields. Thus, we choose the ligand 4,4'- (1,2-dihydroxyethane-1,2-diyl)dibenzoate and Co(NO3)_2_ under hydrothermal conditions to obtain new metal-organic complex, We report here the synthesis and structure of the title compound.

As shown in Figure 1, the title compound [Co(H_2_O)_6_][C_16_H_12_O_6_].H_2_O is composed of one 4,4'-(1,2-dihydroxyethane-1,2-diyl)dibenzoate anion lying on inversion center, one [Co6H_2_O]^2+^ dicationic complex and a solvent water molecule locating on mirror planes. The Co^II^ ion is coordinated by six water molecules in an octahedral geometry. The hydroxyl groups of the 4,4'-(1,2-dihydroxyethane-1,2-diyl)dibenzoate anion are oriented such the the H atoms are directed away from the plane of the benzene ring. In the crystal, a one-dimensional chain is constructed via O—H···O hydrogen bonds interactions involving the carboxylate and hydroxyl groups of the organic anion, which was further connected into a three-dimensional framework by additional O—H···O hydrogen bonds formed by [Co(H_2_O)_6_]^2+^ cations, solvent water molecules and the anions.

### Experimental

A mixture of 4,4'-(1,2-dihydroxyethane-1,2-diyl)dibenzoate(0.5 mol, 0.15 g) and Co(NO_3_)_2_ (0.5 mol, 0.14 g) in 30 ml of absolute ethanol was heated under reflux for 6 h in the presence of 1-2 drops of NaOH. The reaction mixture was cooled to room temperature for 2 h. The light-red crystal was filtered off and washed several times using absolute ethanol.

### Refinement

H atoms bound to C atoms were placed at calculated positions and were treated as riding on the parent atoms, with C—H = 0.93 Å(aromatic) and 0.98 Å(CH) and with *U*_iso_(H) = 1.2 *U*_eq_(C) H atoms of hydroxyl group and water molecules were located in a difference Fourier map and refined as riding, with O—H = 0.85 Å and *U*_iso_(H) = 1.5 *U*_eq_(O) for water O atoms and 1.2 *U*_eq_(O) for hydroxyl O atoms.

### Crystal data

**Table:**

[Co(H_2_O)_6_](C_16_H_12_O_6_)·H_2_O	*F*(000) = 506
*M**_r_* = 485.30	*D*_x_ = 1.555 Mg m^−^^3^
Monoclinic, *P*2_1_/*m*	Mo *K*α radiation, λ = 0.71073 Å
Hall symbol: -P 2yb	Cell parameters from 2215 reflections
*a* = 6.0430 (6) Å	θ = 2.5–24.0°
*b* = 20.487 (2) Å	µ = 0.89 mm^−^^1^
*c* = 8.6341 (9) Å	*T* = 298 K
β = 104.115 (1)°	Block, red
*V* = 1036.66 (18) Å^3^	0.38 × 0.20 × 0.18 mm
*Z* = 2	

### Data collection

**Table:**

Bruker SMART 1000 CCD area-detector diffractometer	1867 independent reflections
Radiation source: fine-focus sealed tube	1675 reflections with *I* > 2σ(*I*)
graphite	*R*_int_ = 0.039
φ and ω scans	θ_max_ = 25.0°, θ_min_ = 2.0°
Absorption correction: multi-scan (*SADABS*; Bruker, 2007)	*h* = −6→7
*T*_min_ = 0.728, *T*_max_ = 0.856	*k* = −24→24
5184 measured reflections	*l* = −8→10

### Refinement

**Table:**

Refinement on *F*^2^	Secondary atom site location: difference Fourier map
Least-squares matrix: full	Hydrogen site location: inferred from neighbouring sites
*R*[*F*^2^ > 2σ(*F*^2^)] = 0.075	H-atom parameters constrained
*wR*(*F*^2^) = 0.177	*w* = 1/[σ^2^(*F*_o_^2^) + (0.*P*)^2^ + 7.8675*P*] where *P* = (*F*_o_^2^ + 2*F*_c_^2^)/3
*S* = 1.25	(Δ/σ)_max_ < 0.001
1867 reflections	Δρ_max_ = 0.58 e Å^−^^3^
144 parameters	Δρ_min_ = −0.46 e Å^−^^3^
11 restraints	Extinction correction: *SHELXL*, Fc^*^=kFc[1+0.001xFc^2^λ^3^/sin(2θ)]^-1/4^
Primary atom site location: structure-invariant direct methods	Extinction coefficient: 0.0093 (18)

### Special details

**Table:**

Geometry. All esds (except the esd in the dihedral angle between two l.s. planes) are estimated using the full covariance matrix. The cell esds are taken into account individually in the estimation of esds in distances, angles and torsion angles; correlations between esds in cell parameters are only used when they are defined by crystal symmetry. An approximate (isotropic) treatment of cell esds is used for estimating esds involving l.s. planes.
Refinement. Refinement of F^2^ against ALL reflections. The weighted R-factor wR and goodness of fit S are based on F^2^, conventional R-factors R are based on F, with F set to zero for negative F^2^. The threshold expression of F^2^ > 2sigma(F^2^) is used only for calculating R-factors(gt) etc. and is not relevant to the choice of reflections for refinement. R-factors based on F^2^ are statistically about twice as large as those based on F, and R- factors based on ALL data will be even larger.

### Fractional atomic coordinates and isotropic or equivalent isotropic displacement parameters (Å^2^)

**Table:**

	*x*	*y*	*z*	*U*_iso_*/*U*_eq_	
Co1	0.8509 (2)	0.2500	0.54019 (15)	0.0234 (4)	
O1	0.4205 (10)	0.1418 (3)	−0.2097 (7)	0.0505 (16)	
O2	0.1731 (10)	0.1522 (3)	−0.0588 (8)	0.0616 (19)	
O3	0.8284 (10)	−0.0725 (3)	0.4766 (7)	0.0536 (17)	
H3	0.7734	−0.0959	0.3999	0.080*	
O1W	0.8192 (10)	0.1750 (3)	0.6947 (7)	0.062 (2)	
H1W	0.6984	0.1651	0.7232	0.093*	
H2W	0.9330	0.1678	0.7727	0.093*	
O2W	0.9112 (10)	0.1813 (3)	0.3752 (7)	0.0540 (16)	
H3W	0.9892	0.1489	0.4198	0.081*	
H4W	0.9848	0.2002	0.3158	0.081*	
O3W	0.5046 (14)	0.2500	0.4420 (9)	0.061 (3)	
H5W	0.4269	0.2500	0.5118	0.091*	
H6W	0.4125	0.2500	0.3502	0.091*	
O4W	1.2136 (12)	0.2500	0.6463 (9)	0.0351 (17)	
H7W	1.2522	0.2875	0.6825	0.053*	
O5W	0.1686 (19)	0.2500	0.1613 (12)	0.099 (4)	
H9W	0.1690	0.2164	0.1036	0.148*	
C1	0.3585 (14)	0.1323 (4)	−0.0814 (10)	0.044 (2)	
C2	0.5138 (13)	0.0948 (4)	0.0530 (10)	0.041 (2)	
C3	0.7192 (14)	0.0687 (4)	0.0317 (10)	0.045 (2)	
H3A	0.7632	0.0762	−0.0627	0.054*	
C4	0.8566 (15)	0.0316 (4)	0.1527 (10)	0.047 (2)	
H4	0.9921	0.0142	0.1380	0.056*	
C5	0.7956 (14)	0.0202 (4)	0.2936 (10)	0.043 (2)	
C6	0.5928 (15)	0.0463 (4)	0.3163 (10)	0.048 (2)	
H6	0.5508	0.0393	0.4117	0.058*	
C7	0.4528 (14)	0.0831 (4)	0.1944 (10)	0.047 (2)	
H7A	0.3163	0.0999	0.2089	0.056*	
C8	0.9495 (15)	−0.0203 (4)	0.4267 (10)	0.046 (2)	
H8A	1.0745	−0.0386	0.3863	0.055*	

### Atomic displacement parameters (Å^2^)

**Table:**

	*U*^11^	*U*^22^	*U*^33^	*U*^12^	*U*^13^	*U*^23^
Co1	0.0209 (7)	0.0289 (7)	0.0194 (7)	0.000	0.0032 (5)	0.000
O1	0.041 (3)	0.049 (4)	0.053 (4)	0.002 (3)	−0.005 (3)	0.010 (3)
O2	0.039 (4)	0.075 (5)	0.062 (4)	0.016 (3)	−0.005 (3)	0.024 (4)
O3	0.058 (4)	0.037 (3)	0.054 (4)	−0.004 (3)	−0.010 (3)	0.004 (3)
O1W	0.035 (3)	0.086 (5)	0.058 (4)	−0.008 (3)	−0.002 (3)	0.033 (4)
O2W	0.056 (4)	0.053 (4)	0.050 (4)	0.000 (3)	0.006 (3)	−0.010 (3)
O3W	0.037 (5)	0.118 (8)	0.024 (4)	0.000	−0.001 (4)	0.000
O4W	0.033 (4)	0.031 (4)	0.039 (4)	0.000	0.006 (3)	0.000
O5W	0.068 (7)	0.184 (14)	0.046 (6)	0.000	0.017 (5)	0.000
C1	0.039 (5)	0.038 (5)	0.047 (5)	−0.006 (4)	−0.009 (4)	0.008 (4)
C2	0.036 (4)	0.030 (4)	0.045 (5)	−0.001 (3)	−0.011 (4)	0.004 (4)
C3	0.045 (5)	0.039 (5)	0.044 (5)	0.001 (4)	−0.003 (4)	0.007 (4)
C4	0.042 (5)	0.041 (5)	0.047 (5)	0.011 (4)	−0.008 (4)	0.004 (4)
C5	0.040 (5)	0.029 (4)	0.047 (5)	−0.001 (3)	−0.014 (4)	0.002 (4)
C6	0.044 (5)	0.050 (5)	0.041 (5)	−0.001 (4)	−0.007 (4)	0.008 (4)
C7	0.034 (4)	0.045 (5)	0.054 (5)	0.002 (4)	−0.003 (4)	0.009 (4)
C8	0.044 (5)	0.032 (4)	0.048 (5)	0.000 (4)	−0.013 (4)	0.006 (4)

### Geometric parameters (Å, °)

**Table:**

Co1—O3W	2.058 (8)	O4W—H7W	0.8413
Co1—O1W^i^	2.074 (6)	O5W—H9W	0.8500
Co1—O1W	2.074 (6)	C1—C2	1.511 (10)
Co1—O2W	2.097 (6)	C2—C7	1.380 (12)
Co1—O2W^i^	2.097 (6)	C2—C3	1.404 (12)
Co1—O4W	2.159 (7)	C3—C4	1.392 (11)
O1—C1	1.268 (10)	C3—H3A	0.9300
O2—C1	1.251 (11)	C4—C5	1.374 (12)
O3—C8	1.420 (10)	C4—H4	0.9300
O3—H3	0.8200	C5—C6	1.394 (12)
O1W—H1W	0.8500	C5—C8	1.534 (10)
O1W—H2W	0.8500	C6—C7	1.399 (11)
O2W—H3W	0.8500	C6—H6	0.9300
O2W—H4W	0.8500	C7—H7A	0.9300
O3W—H5W	0.8501	C8—C8^ii^	1.513 (16)
O3W—H6W	0.8500	C8—H8A	0.9800
			
O3W—Co1—O1W^i^	91.2 (2)	O2—C1—C2	117.2 (8)
O3W—Co1—O1W	91.2 (2)	O1—C1—C2	119.3 (8)
O1W^i^—Co1—O1W	95.6 (4)	C7—C2—C3	119.0 (7)
O3W—Co1—O2W	92.7 (2)	C7—C2—C1	121.2 (8)
O1W^i^—Co1—O2W	173.2 (3)	C3—C2—C1	119.7 (8)
O1W—Co1—O2W	89.9 (3)	C4—C3—C2	119.6 (8)
O3W—Co1—O2W^i^	92.7 (2)	C4—C3—H3A	120.2
O1W^i^—Co1—O2W^i^	89.9 (3)	C2—C3—H3A	120.2
O1W—Co1—O2W^i^	173.2 (3)	C5—C4—C3	121.2 (8)
O2W—Co1—O2W^i^	84.3 (4)	C5—C4—H4	119.4
O3W—Co1—O4W	179.3 (3)	C3—C4—H4	119.4
O1W^i^—Co1—O4W	88.3 (2)	C4—C5—C6	119.6 (7)
O1W—Co1—O4W	88.3 (2)	C4—C5—C8	120.7 (8)
O2W—Co1—O4W	87.8 (2)	C6—C5—C8	119.7 (8)
O2W^i^—Co1—O4W	87.8 (2)	C5—C6—C7	119.5 (8)
C8—O3—H3	109.5	C5—C6—H6	120.3
Co1—O1W—H1W	125.7	C7—C6—H6	120.3
Co1—O1W—H2W	117.0	C2—C7—C6	121.1 (8)
H1W—O1W—H2W	108.4	C2—C7—H7A	119.4
Co1—O2W—H3W	112.7	C6—C7—H7A	119.4
Co1—O2W—H4W	107.9	O3—C8—C8^ii^	107.0 (9)
H3W—O2W—H4W	108.4	O3—C8—C5	111.8 (7)
Co1—O3W—H5W	112.9	C8^ii^—C8—C5	112.1 (8)
Co1—O3W—H6W	138.9	O3—C8—H8A	108.6
H5W—O3W—H6W	108.2	C8^ii^—C8—H8A	108.6
Co1—O4W—H7W	108.6	C5—C8—H8A	108.6
O2—C1—O1	123.5 (8)		
			
O2—C1—C2—C7	0.1 (12)	C4—C5—C6—C7	0.7 (12)
O1—C1—C2—C7	−179.7 (8)	C8—C5—C6—C7	−179.9 (7)
O2—C1—C2—C3	177.1 (8)	C3—C2—C7—C6	0.6 (13)
O1—C1—C2—C3	−2.8 (12)	C1—C2—C7—C6	177.5 (7)
C7—C2—C3—C4	0.1 (12)	C5—C6—C7—C2	−1.0 (13)
C1—C2—C3—C4	−176.9 (7)	C4—C5—C8—O3	−126.6 (9)
C2—C3—C4—C5	−0.4 (13)	C6—C5—C8—O3	54.0 (10)
C3—C4—C5—C6	0.0 (13)	C4—C5—C8—C8^ii^	113.3 (11)
C3—C4—C5—C8	−179.4 (7)	C6—C5—C8—C8^ii^	−66.1 (12)

Symmetry codes: (i) *x*, −*y*+1/2, *z*; (ii) −*x*+2, −*y*, −*z*+1.

### Hydrogen-bond geometry (Å, °)

**Table:**

*D*—H···*A*	*D*—H	H···*A*	*D*···*A*	*D*—H···*A*
O3—H3···O1^iii^	0.82	2.00	2.811 (8)	168
O1W—H1W···O1^iv^	0.85	1.96	2.814 (9)	180
O1W—H2W···O2^v^	0.85	1.81	2.665 (8)	179
O2W—H3W···O3^ii^	0.85	2.00	2.847 (8)	180
O2W—H4W···O5W^vi^	0.85	2.19	3.035 (11)	179
O3W—H5W···O4W^vii^	0.85	1.93	2.778 (11)	172
O3W—H6W···O5W	0.85	1.91	2.756 (13)	171
O5W—H9W···O2	0.85	1.93	2.767 (10)	169
O4W—H7W···O1^viii^	0.84	1.88	2.695 (7)	163
O4W—H7W···O2^viii^	0.84	2.70	3.296 (9)	130

Symmetry codes: (iii) −*x*+1, −*y*, −*z*; (iv) *x*, *y*, *z*+1; (v) *x*+1, *y*, *z*+1; (ii) −*x*+2, −*y*, −*z*+1; (vi) *x*+1, *y*, *z*; (vii) *x*−1, *y*, *z*; (viii) *x*+1, −*y*+1/2, *z*+1.
